# A novel edge-marking method in pleural covering with video-assisted thoracic surgery

**DOI:** 10.1093/icvts/ivad046

**Published:** 2023-03-13

**Authors:** Aki Fujiwara-Kuroda, Yasuhiro Hida, Hideki Ujiie, Kichizo Kaga, Tatsuya Kato

**Affiliations:** Department of Thoracic Surgery, Hokkaido University Hospital, Sapporo, Japan; Department of Thoracic Surgery, Hokkaido University Hospital, Sapporo, Japan; Department of Thoracic Surgery, Hokkaido University Hospital, Sapporo, Japan; Department of Thoracic Surgery, Hokkaido University Hospital, Sapporo, Japan; Department of Thoracic Surgery, Hokkaido University Hospital, Sapporo, Japan

**Keywords:** Pleural covering, Lymphangioleiomyomatosis, Reduced port video-assisted thoracic surgery, Covering technique, Oxidized cellulose mesh

## Abstract

Total pleural covering is implemented to reinforce the visceral pleura with surgical sheets. It has been adopted for diffuse cystic lung diseases such as lymphangioleiomyomatosis to prevent pneumothorax and has achieved good results. The procedure is technically demanding, because it is difficult to cover the entire visceral pleura without disarrangement and jamming of surgical sheets, especially during thoracoscopic surgery, where grasping of a wrong site might happen when unfolding the sheets. Herein, we report a technique to cover the entire pleura with dotted line folded sheets to ease the thoracoscopic procedure. We found that the use of this marking method made the procedure easier, with just a little ingenuity, because marking the edges of sheets with dashed lines clarifies the site that should be grasped, thus preventing the incidence of grasping the wrong part of the sheet. Pleural covering with dotted line folded surgical sheets is a useful method for reduced port thoracoscopic surgery.

## INTRODUCTION

Pleural covering using regenerative oxidized cellulose mesh is a surgical procedure devised to prevent the recurrence of diffuse cystic lung disease such as lymphangioleiomyomatosis (LAM). Total pleural covering (TPC) is useful because it can prevent the recurrence of pneumothorax without the risk of pleurodesis. Furthermore, because of its usefulness, the indication for pleural covering is expanding to other pneumothorax than cystic lung disease. However, it is difficult to cover the entire pleura with pre-folded mesh sheets without disarrangement and jamming during video-assisted thoracic surgery (VATS). Herein, we describe a unique method to ensure the order of unfolding by marking the edges of the pre-folded mesh sheets.

## TECHNIQUE

**Figure 1:**
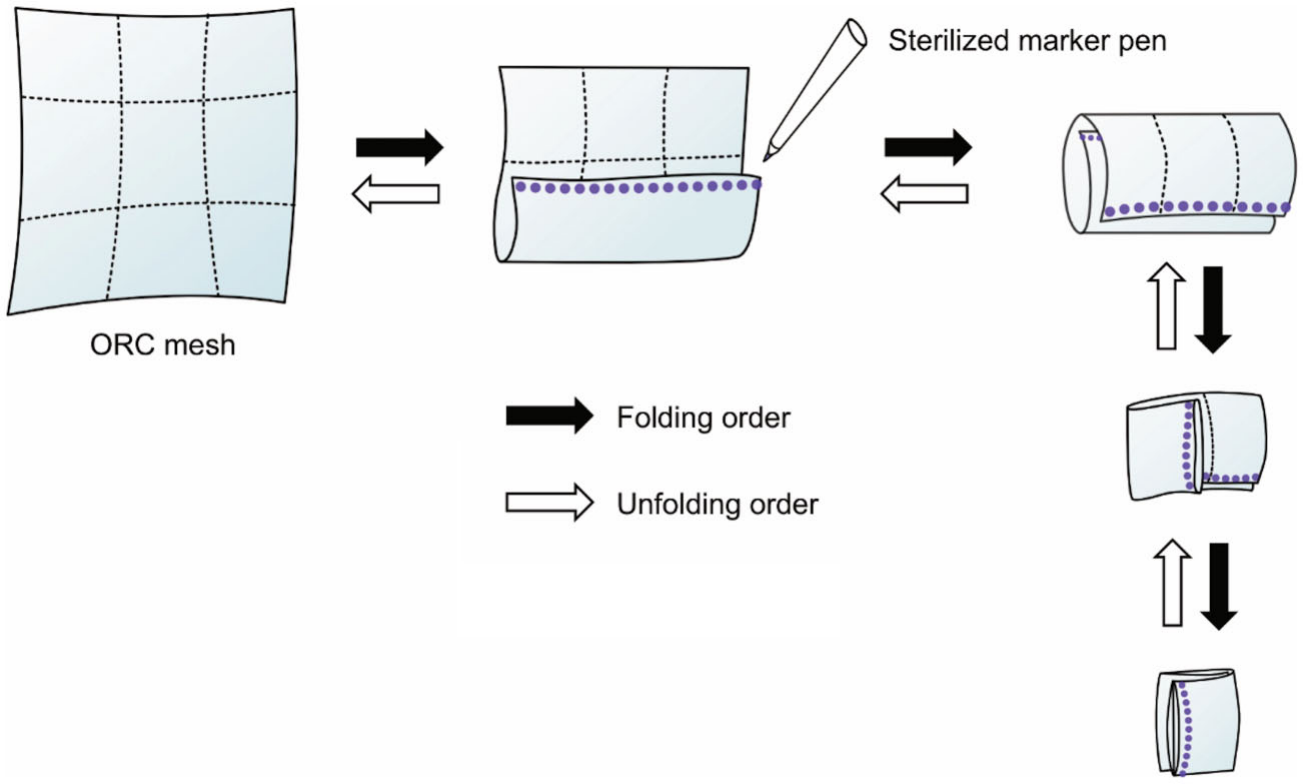
Schema of marking the edge of an ORC mesh. First, fold the 10.2 cm × 20.3 cm-sized mesh into 9 equal parts. Second, check the order of unfolding before inserting the mesh into the thoracic cavity, and mark the edges that need to be grasped with a dashed line using a sterilized marker pen (it is also important not to mark unnecessary edges). The black block arrows and white arrows indicate the folding and unfolding order, respectively. ORC: oxidized regenerated cellulose

First, fold the 10.2 cm × 20.3 cm-sized mesh into 9 equal parts, similar to how it is pre-folded and packed. Second, check the order of unfolding and mark the edges that need to be grasped with a dashed line using a sterilized marker pen. Third, insert the mesh into the thoracic cavity and place it at the centre of the target position on the lung surface. Finally, unfold the mesh in the scheduled order by grasping the marked edge (Fig. 1 and Video 1). This process must be performed as quickly as possible, because oxidized regenerated cellulose (ORC) meshes become increasingly difficult to handle as time passes after opening since they absorb moisture from the air and the lung.

We performed 6 pleural covering procedures by thoracoscopic surgery in 5 patients with LAM, idiopathic pulmonary hemosiderosis, or spontaneous pneumothorax (Table 1). Among them, the new folding method was used in the latter 4. We performed 1 case with uniportal VATS and 4 cases with two-port VATS. Although there was no decrease in operative time with our marking method, the covering time per sheet measured by dividing covering time by the number of sheets, tended to decrease in the latter cases with our marking method. No relapse of pneumothorax has occurred in any of the cases after surgery. All patients gave informed consent for the procedure and for the presentation of the data.

**Table 1:** Operative features of the 4 pleural covering procedures performed in 3 patients

Case	Diagnosis	Side	Preoperative PTX frequency	Total/partial covering	Number of ports	Number of sheets [*a*]	OT (min)	Minute for covering [*b*]	Covering time/sheet [*b*/*a*] (min)	Marking method
1	LAM	Right	1	Total	3	7	97	0:41:32	5:56	None
2–1	LAM	Right	2	Total	2	7	131	0:49:14	7:02	Line
2–2	LAM	Left	2	Total	2	9 and 1/3	154	1:05:00	6:58	Dashed line
3	IPH	Right	1	Partial	1	5	108	0:27:52	5:34	Dashed line
4	SP	Left	1	Total	2	12	153	1:04:11	5:21	Dashed line
5	SP	Right	2	Partial	2	5	182	0:19:40	3:56	Dashed line

IPH: idiopathic pulmonary hemosiderosis; LAM: lymphangioleiomyomatosis; OT: operation time; PTX: pneumothorax; SP: spontaneous pneumothorax.

Our folding method, named dotted line folded sheet procedure, is a novel method of marking the edges of the folded mesh sheets to identify the order of the unfolding process after their insertion into the pleural cavity. This procedure helps to identify the edges of the sheets easily, unfold them quickly and prevent unnecessary detachment or jamming of the sheet in the limited visibility during VATS.

## COMMENT

Kurihara *et al.* [1] reported TPC as a method of covering the entire visceral pleura with ORC-mesh sheets and fibrin glue to prevent the recurrence of pneumothorax. TPC is beneficial for diseases eventually requiring lung transplantation in the future including LAM or BHD syndrome, because it does not cause adhesions between the lung and parietal pleura, while the pleurodesis therapy for the treatment of recurrent pneumothorax can lead to excessive bleeding during transplantation surgery and postoperative morbidity [2]. Pleural covering is a method of preventing the rupture of a new bra by thickening the visceral pleura, not by causing adhesion to the parietal pleura. This is the reason why this procedure reduces the incidence of bleeding during lung transplantation; however, postoperative prolonged air leakage can be a problem. In recent years, the indications for TPC have been expanded from only LAM of the lung to other recurrent pneumothorax cases associated with Birt–Hogg–Dubé syndrome, eosinophilic pneumonia and spontaneous pneumothorax [3–6]. In particular, regenerated bullae at the edge of the suture line after bullectomy often contribute to postoperative recurrence of spontaneous pneumothorax; the visceral pleura should be a target when treating or preventing a recurrence of pneumothorax [6]. It is supposed that the adhesions around the suture line may pull the regenerated bulla, causing a recurrence or delay in healing after surgery. Thus, pleural covering is also useful as a method of preventing postoperative recurrence of spontaneous pneumothorax. Considering the expansion of the pleural covering from rare diseases such as LAM to more common diseases such as recurrent spontaneous pneumothorax, we believe that thoracic surgeons need to become adept at this procedure.

The procedure is technically demanding, because it is difficult to cover the entire visceral pleura without jamming the surgical sheets, especially during thoracoscopic surgery. As the sheets overlap, the edge of the sheet becomes unclear, and it becomes difficult to unfold the meshes. We found that the use of dotted line folded surgical sheets made the procedure easier (Fig. 1 and Video 1). Marking the edges of the sheets with dashed lines clarifies the site that should be grasped and thus helps to avoid grasping the wrong part of the sheet. We scrapped the use of solid line marking, as it darkened the thoracoscopic view. The advantage of this method is that it can be performed with very simple preparation. Moreover, it can be performed quickly before the ORC mesh absorbs water and becomes difficult to handle.

Several large mesh sheets are required to be inserted into the pleural cavity through a small incision to cover the entire pleura without gaps. Therefore, it is easier to divide each sheet into several pieces before inserting it into the thoracic cavity. However, it can be beneficial to use entire sheets without division because divided sheets are more likely to create uncovered gap areas when the lungs expand.

Pleural covering has been performed with three- or four-port VATS; however, reduced port surgery is cosmetically more beneficial, especially among younger patients. We completed pleural covering with reduced port VATS by adopting the dotted line folded surgical sheet method. Although this is not a comparative study and it has not proven the benefit of our new method, it may be useful for performing pleural covering with reduced port VATS.

We found that pleural covering with dotted line folded surgical sheets is a very simple but useful method and well worth trying when performing pleural covering with reduced port VATS.
